# A preliminary study of skin bleaching and factors associated with skin bleaching among women living in Zimbabwe

**DOI:** 10.4314/ahs.v21i1.18

**Published:** 2021-03

**Authors:** Princess Nyoni-Kachambwa, Wanapa Naravage, Nigel F James, Marc Van der Putten

**Affiliations:** Faculty of Public Health, Thammasat University Rangsit Campus, Khlong 1, Khlong Luang, Pathumthani, Thailand

**Keywords:** Skin bleaching, skin bleaching products, women, Zimbabwe

## Abstract

**Background:**

Skin bleaching was reported to be commonly practiced among women and Africa was reported to be one of the most affected yet the subject is not given much attention in public health research in Zimbabwe despite the adverse effects of skin bleaching on health.

**Method:**

This study was an exploratory cross-sectional survey to explore skin bleaching, skin bleaching patterns and factors associated with skin bleaching among women living in Zimbabwe. An online self-administered questionnaire was sent out to women on social network i.e. WhatsApp, Facebook, LinkedIn and Twitter.

**Findings:**

A total number of 260 respondents, mean age 31.69 (SD, 8.12) years participated in the survey. The prevalence of skin bleaching among the participants was 31.15%. The major reason reported for skin bleaching was to have smooth and healthy skin alongside other factors such as beauty, gaining social favours for example getting married and good jobs. Occupation, complexion and marital status were associated with skin bleaching. The odds of skin bleaching for participants who were employed was 1.45(95% confidence interval [CI],0.32–1.91);p-value 0.02, dark skinned participants 2.56(95% CI, 0.76–2.87);p-value 0.01 and unmarried participants 2.87(95% CI,0.29–3.58);p-value 0.03.

**Conclusion:**

Evidence from the research suggests skin bleaching might be common among women living in Zimbabwe and possibly poses serious health threats to the women. Skin bleaching seems to be deep rooted in colourism. The colourism seems to be taken advantage of by the cosmetic industry which produce the potentially hazardous products which promise the revered light skin to women but which comes with a price. However, the study provides a base for future studies to explore more on skin bleaching practices among women living in Zimbabwe.

## Introduction

Despite its potentially adverse effects, skin bleaching has reached epidemic levels around the globe. Skin bleaching is generally the lightening of the skin and is typically acceptable for medicinal purposes such as depigmentation of darker parts of the body for example age or acne sports. However, most people are bleaching their skin for cosmetic purposes. Although some of the studies' findings might not have been representative enough due to reasons such as convenience sampling, skin bleaching for cosmetics purposes was reported to be high and most common among women in Africa^1^,^2^,^3^. The practice was also reported in other regions such as some Asian countries^4^,^17^, some populations in America such as Caribbean born blacks and Dominicans^26^ and some countries in Europe^27^. In Zimbabwe, a prevalence of 20% among university students was reported^5^ but nothing was identified in the general women population. However, anecdotal evidence from non-academic sources imply skin bleaching could be highly practiced by women in Zimbabwe 6, 7, 8.

Skin bleaching seems to be stemming from colourism. Colourism is the discrimination of people due to their skin colour in which the light skin is revered. This reverent of light skin has given light skinned people an advantage for example good jobs in some societies 9, 10. Cosmetic industries have therefore been capitalizing on that colourism, making billions by producing skin bleaching cosmetics which promise the valued light skin to consumers, however most of the cosmetics are hazardous. Most of the skin bleaching creams were reported to contain hazardous chemicals for instance hydroquinone and mercury 11, 12, 13. Due to the harmful chemicals in the products, some were reported to cause problems such as exogenous ochronosis (a disorder characterized by a blue-black discoloration on the skin due to prolonged skin bleaching), weakening wound healing and exacerbating kidney problems 14. The effects can be worsened by sun exposure. Direct sun exposure of especially light skin is a risk factor for developing conditions such as melanoma (a severe form of skin cancer) 15. Therefore, directly exposing bleached skin to sunlight could also increase the odds of developing melanoma.

The previously reviewed studies reveal skin bleaching to be common in Africa especially among women. However, the subject is less explored in Zimbabwe. The previously identified studies in Zimbabwe focused on women concentrated in particular areas, for example university students and women in Masvingo province^5^,^16^. Additionally, none of them explored the possible factors associated with skin bleaching among the women. It was therefore of utmost importance to explore further skin bleaching patterns and the possible factors associated with skin bleaching among the women living in Zimbabwe targeting the general women population. The study's main aim was to provide preliminary evidence of skin bleaching practices and possible factors associated with skin bleaching among women living in Zimbabwe to in turn inform future bigger studies.

## Methodology

### Study design and procedure

An explorative cross-sectional online survey was conducted among 260 women living in Zimbabwe. Participants were conveniently selected through an online survey to complete online questionnaires.

### Study participants and sampling

The target population was women either Zimbabweans or non-Zimbabweans, 18 years and above living in Zimbabwe. Participants were conveniently selected online via social networks i.e. WhatsApp, Facebook, LinkedIn and Twitter. The questionnaire link was sent on the social networks to as many as possible women in Zimbabwe so as to try and increase coverage of the study and its precision. The questionnaire link was sent to the participants using Survey Monkey (an online survey software). Participants who gave consent proceeded with the survey. Participants were not given any compensation for participating in the study.

### Inclusion and exclusion criteria

Women who lived in Zimbabwe and of any nationality, who were 18+ years, who had internet access, who participated on social networks i.e. Facebook, WhatsApp, LinkedIn and Twitter plus who were willing to give consent were included in the study. The participants were asked where they were currently residing at the time of the study and were to choose from 2 options; Zimbabwe and other. There were follow up questions to further verify the participants' residence. The questions additional asked the women who had reported to be out of Zimbabwe how long they have been out of Zimbabwe and the reasons for leaving Zimbabwe. If the women had reported to be out of Zimbabwe for over 6 months and out of Zimbabwe for any reason except short visit, were excluded from the study. Women who had reported to be living in Zimbabwe were further asked their reasons for being in Zimbabwe. The women who reported to be in Zimbabwe just for short visits were also excluded from the study. Women who possessed all the qualities for inclusion but did not live in Zimbabwe i.e. who had just visited Zimbabwe, who had participated in the pretesting of the questionnaire and those who were not willing to give consent were excluded from the study. The questionnaire was not sent to the women who had participated in the pretesting of the questionnaire. Participants who were not willing to give consent and below 18 years were automatically excluded from the study which was enabled through the data collection software settings whilst the rest were excluded after data collection on the basis of their responses to questions which enabled them inclusion.

### Study instruments and measures

Online self-administered questionnaires were distributed to the women using Survey Monkey. The questionnaire was structured into 5 sections;

- Demographic data of the participants section: assessed women's marital status, age, religion, occupation, education level, their skin tone and their locations.
- Skin bleaching patterns and knowledge on skin bleaching side effects section: finding out how many women were bleaching their skin, how many would consider skin bleaching in future, how they bleach their skin, how they frequently apply skin bleaching products, assessing whether they know the side effects of skin bleaching.
- Exposure, accessibility and cost of skin bleaching products section: finding out how they got to know about skin bleaching products, where they purchase the products and the cost of the skin bleaching products.
- Reasons for skin bleaching section: assessing why the women were bleaching their skin.
- Associations of skin bleaching and various factors section: finding out what possible demographic factors were related to skin bleaching.

The questions were closed ended. After doing a considerable literature review, the research questions and objectives of the study were formed based on the review. The questionnaire was formed based on the research objectives. The questionnaire went through content validity by giving it to 2 experts who provided feedback on how well the questions were measuring the study's constructs. The operational definition of skin bleaching in the study was the lightening of the skin for cosmetic purposes.

### Pretesting of questionnaires

The questionnaire was pretested to 50 women residing in Zimbabwe at the time of the study, whom the researcher knew in person i.e. colleagues and friends. The questionnaire was sent to the researcher's colleagues and friends through email and WhatsApp. The questionnaire was pretested to already known women so that it would be easier to exclude them from the study. The questionnaire was pretested to identify unclear questions and to assess whether the online survey was easy to use. In addition, the questionnaire was given to 2 experts in survey research to analyse the appropriateness of the items to establish content validity.

### Ethical aspects

The study was approved by Thammasat University, Ethical board (approval code-3: ECScTU). A written online consent form was also attached to the online questionnaire which disclosed all the details of the study to the participants. The consent form was designed in such a way that the participants could not proceed to the questionnaire without giving consent. Participation was based on volunteering and participants were not allowed to put their personal details on the questionnaire so as to privatise their information.

### Data analysis

Data were collected from 1 May 2017 to 4 August 2017 including pretesting of the questionnaire. The collected data was exported from survey monkey software to Statistical Package for the Social Sciences (SPSS) version 23 which was used to analyse the data. The data was first cleaned. A quantitative descriptive analysis was performed on the questionnaires. Binary logistic regression was further performed on the questionnaires to ascertain relationship between the demographic factors and skin bleaching.

## Results

### Demographic data of the participants

All the participants reported to be Zimbabweans. The mean age of the women was 31.69(Standard Deviation, 8.12) years. The median age was 31 (Interquartile Range, 27–37) years. Age was grouped into 3 categories; young adults which were grouped between 18–35 years, middle aged adults from 36–50 years and mature adults to older women over 50 years. Participants were asked to choose a category for their skin tone out of 4 categories; very light, light, medium brown and dark brown. The data from the categories were recoded into 2 categories for ease of analysis; the light and very light as light skinned and the other 2 as dark skinned. Table 1 clearly illustrates the demographic characteristics of the participants.

**Table 1:** Demographic characteristics of participants (n=260)

		Frequency	%Frequency
**Marital Status**	Married	129	49.62
	Single	103	39.62
	Others (divorced & widowed)	28	10.77

**Occupation**	Student	48	18.46
	Employed	141	54.23
	Not employed	71	27.31

**Education level**	Primary	1	0.38
	Secondary	22	8.46
	Tertiary	237	91.15

**Religion**	Christians	235	90.38
	Muslim	3	1.15
	ATR	2	0.77
	No religion	20	7.69

**Age**	Young Adults	200	76.92
	Middle aged adults	51	19.62
	Mature adults to	9	3.46
	the elderly		
**Skin tone**	Light skinned	68	26.15
	Dark skinned	192	73.85

**Location**	Urban	191	73.46
	Rural	69	26.54

### Skin Bleaching patterns and knowledge on skin bleaching side effects

Participants who reported to be bleaching their skin were 81 giving a prevalence of skin bleaching of 31.15% in the study. Of the women who did not bleach their skin, 36% reported that they would consider skin bleaching given that the side effects of skin bleaching were very minimal. Just above half of the women (52.31%) who reported to bleach their skin admitted they knew about the side effects of skin bleaching. Of the women using skin lightening agents, 92.59% reported to be applying them topically and the rest used injections and tablets. A total of 66.61% of those who applied skin bleaching products topically reported to be applying the products at-least twice daily. All the participants who reported to be using injections and tablets reported to be using them once monthly and once every day respectively. Sunscreen use was reported to be low with only 27.16 % of the participants reporting to be using it when exposed to the sun.

### Exposure to skin bleaching, accessibility and cost of skin bleaching products

The average cost of the skin bleaching products was 32. 27(SD, 74.24) United States (US) dollars. Modal cost of products was 15 US dollars. The participants were asked to rate affordability of general cosmetic products on a likert scale, so that it could be estimated how expensive were the skin bleaching products. Based on the scale, 69.80% participants thought that cosmetic products which costed 10 US dollars and below were affordable whilst 72.87% thought that cosmetic products which costed over 10 US dollars were expensive. Majority of the participants were introduced to skin bleaching by advertisements (72.84%) whilst the rest reported to have been influenced by colleagues. Most of the products were reported to be purchased in shops (51.09%) in streets (31.32%), pharmacy (11.42%) and online shops (6.17%).

### Women's reasons for skin bleaching

The women were further asked their reasons for skin bleaching. One of the reasons for skin bleaching was to possess smooth and healthy skin as reported by 49.38 %, to look beautiful as reported by 30.86% and the rest of the women reported to obtain social favours such as marriage and good jobs.

### Association of skin bleaching and various factors

Logistic regression was further run on the data. Logistic regression was used to estimate whether some of the independent variables i.e. demographics had an association with skin bleaching and to estimate the magnitude of the association. The regression model yielded a significant p-value of 0.03 on the Omnibus Test and an insignificant value of 0.34 on Hosmer and lemeshow test and it had a 68.80 percent ability of predicting correctly which made it a relative good model. All the variables were dummy coded into 2 categories for the regression analysis. The predicted probability was of the membership of the yes category for the dependent variable i.e. the category for skin bleachers. Table 2 will display the findings.

**Table 2:** Binary logistic regression (n=260)

Variable	B	S.E.	Wald	P-value	Adjusted OR (95% CI)
**Age**	0.23	0.51	0.78	0.51	1.63(0.43–2.06)
**Education** **level**	-0.57	0.33	3.68	0.18	0.31(0.27–1.37)
**Skin tone**	0.87	0.39	6.56	0.01*	2.56(0.76–2.87)
**Occupation**	0.51	0.46	1.34	0.02*	1.45(0.32–1.91)
**Marital status**	0.89	0.63	3.93	0.03*	2.87(0.29–3.58)
**Religion**	0.32	0.86	0.76	0.21	1.65(0.34–1.78)
**Location**	0.53	0.49	0.96	0.12	1.57(0.34–1.63)

* means statistically significant

Note Abbreviations: OR-odds ratio, CI-confidence interval

### Key for dummy coded variables

Education level; 0= tertiary education, 1= without tertiary education

Complexion; 0= light skinned, 1= dark skinned

Occupation; 0=not employed, 1=employed

Marital Status; 0= married, 1=not married

Religion; 0= non-Christians, 1= Christians

Location;0=rural, 1=urban

Age;0=older adults, 1=young adults

### Occupation and skin bleaching

Employed women had high odds of bleaching their skin. The odds of skin bleaching by employed women were 1.45(95% CI, 0.32–1.91);p-value 0.02 relative to unemployed women.

### Complexion and skin bleaching

The odds of skin bleaching for dark skinned women were 2.56(95% CI, 0.76–2.87);p-value 0.01 compared to light skinned women.

### Marital Status and skin bleaching

Being single, divorced, or being widowed was associated with skin bleaching whereby the odds of skin bleaching for the women in the previously mentioned category was 2.87(CI,0.29–3.58);p-value 0.03 in relation to married women.

### Religion, location, education level and age

Religion, age, location and education level of the women did not have any significant effect on skin bleaching.

## Discussion

The study found 81 women to have been bleaching their skin giving a prevalence of just above 30 percent which was similarly reported in other areas such as South Africa^17^. A third of the participants using skin lightening products suggest skin bleaching could be common among women living in Zimbabwe with a possibility of increase since an additional 36 % of the non-skin bleachers reported that they would consider skin bleaching provided the side effects of the products were minimal. Just above 50% of the participants who bleached their skin reported to know about skin bleaching side effects. Continuous awareness against skin bleaching is therefore of utmost importance which do not only focus on educating side effects of skin bleaching since it seems some women know about the side effects but will still choose to bleach their skin. Therefore, we ought as public health professionals to develop comprehensive strategies for the awareness to try and discourage this health threatening practice.

Skin bleaching has a higher potential of posing serious health effects to the women living in Zimbabwe since most of them use skin bleaching products frequently i.e. just over 65 percent applying skin bleaching products at least twice daily and all those who use skin bleaching tablets taking them daily. Most skin bleaching products were reported to contain harmful ingredients for example mercury 18,19 and therefore, frequently applying these products to the body can mean frequently exposing of oneself to these harmful elements which increases the severity of the harm which can be caused by the products. The chemicals in skin bleaching products have been reported to be attributable to some skin conditions such as scabies, eczema, severe acne and more serious conditions could develop as a result of skin bleaching such as skin cancer, liver impairment and diabetes^28^,^29^. Skin bleaching reduces melanin (a pigment which produces colour in human skin). Melanin provides skin with protection from sun damage therefore reduction of melanin by skin bleaching can then expose the skin to harmful sun rays especially if one is not protecting themselves from the sun e.g. by using sun screen. However, since a few participants reported to be using sunscreen (just above 25%) under direct sunlight, there is a possibility that a larger portion of these women who are skin bleaching are most likely to be exposed to the harmful sun rays and might get conditions such as melanoma.

Reasons which were identified to be possibly motivating skin bleaching were to have smooth and healthy skin, to be beautiful and obtain social favours in the society such as marriage and good jobs. The previously mentioned reasons for skin bleaching have been reported in other studies as well for instance some studies conducted in Asia and other African countries 20, 21, 22. Some of these reported reasons for skin bleaching in our study (e.g. to obtain social favours) shows that there might be a social advantage associated with light skin. The possibility that some social advantages are associated with light skin as observed in our study suggests colourism could also be present in Zimbabwe. However, our study was only preliminary and further larger studies can confirm the presence of colourism in Zimbabwe. The cosmetic industry takes advantage of that colourism making skin bleaching cosmetics which promise light skin, charging them more whilst hiding the darker side of these products. General cosmetic products which costed at least US10 were regarded as expensive in our study by the participants yet the modal cost of skin bleaching products was US 15 and the average cost being US 32 which can also confirm that skin bleaching products are expensive.

The cosmetic industry's most powerful tool seems to be its strategy in advertising. The industry further endorses colourism in their adverts depicting light skin as beauty, as the ideal skin tone to enable success and that their products would make one achieve that ideal skin tone^13^, ^23^. Nonetheless, the industry claim effectiveness of their products sometimes without any proper tests of products 24. The industry also does not warn consumers of any possible side effects of their products but blame any possible damage of the skin from their products to the sun 24. The misinformation from the adverts could be the reason why almost half of the participants' reason for skin bleaching was to have smooth and healthy skin when it is actually the opposite. Skin bleaching might enable one's skin to temporarily look beautiful and smooth as reported by some of the participants or to obtain the social favours but it cannot make one's skin healthy. Skin bleaching is actually dangerous to one's skin health and the overall health 14 but the cosmetic industry doesn't talk about the dark side of their skin bleaching cosmetics. Other researchers have reported some cosmetic industries have manipulated medical information to make their products look safe to consumers 25. Public health programmes need to find ways to discourage the colourism culture for instance advocating for dark skinned models and using dark skinned women too as role models. Advocating for policies which regulate adverts and their probable false content could be of use as well and discourage false information regarding skin bleaching cosmetics to reach consumers. It is vital for people to have full information about skin bleaching cosmetics so that they can make informed decisions before they decide to use the products.

Age did not have any significant effect on women's skin bleaching practices in disagreement to other African studies 3, 25. This could be due to the nature of the study which was cross sectional. A study stratified for age could further explore the effect of age on skin bleaching. On the other note, it might mean that any woman regardless of age has 50 % probability of bleaching their skin in Zimbabwe. Dark skinned women were identified to have increased odds of bleaching their skin compared to light skinned women, which is expected that the dark skinned women are prone to lightening their skin. Employed women had elevated odds of bleaching their skin in relation to the ones who were not employed. This could be explained by the expensiveness of the skin bleaching products which can be afforded by employed women naturally screening out women who might not afford the products i.e. unemployed women. Contrarily, due to the likely colourism culture in Zimbabwe mentioned earlier, women who are bleaching their skin could be at a social advantage because of their ‘light skin’ though acquired artificially which could be enabling them to get jobs unlike dark skinned women. The likelihood of colourism could also explain why participants who were not married compared to married participants had higher odds of bleaching their skin to acquire the light skin which would probably enhance their chances of getting a marriage partner.

The major limitation of our study was convenience sampling which might have made the study sample not to be representative enough for the women living in Zimbabwe. Therefore, the study findings cannot be generalized to the women living in Zimbabwe. In addition, the data were collected online which is usually known to yield a low response rate. It can also be difficult to validate some of the responses from online surveys for instance whether the respondent was actually a woman in the case of our study. Therefore, future studies need to incorporate probability sampling techniques and collect data in person so that their results can be more valid and representative enough for the women living in Zimbabwe. Nevertheless, our study is worthwhile in providing preliminary evidence on the skin bleaching patterns and the possible factors which might influence skin bleaching among women living in Zimbabwe.

## Conclusion

Skin Bleaching seems to be a common practice among women living in Zimbabwe and has a potential of posing serious threats to the health of the women. The practice seems to be rooted in colorism. The cosmetic industry is capitalizing on that colourism, producing skin bleaching products which promise the consumers the revered light skin though hiding the potential dangers which result from these products. However, the study was only preliminary but it sets a base for future studies which can explore more on the subject and can be more representative for comprehensive strategies to minimize the use of skin bleaching products.
